# Hydrazine imprinted electrochemical sensor based on cobalt-barium stannate nanoparticles incorporated-functionalized MWCNTs nanocomposite for hydrazine determination in tap water samples

**DOI:** 10.1007/s00604-025-06982-9

**Published:** 2025-02-01

**Authors:** Fatma Hazan Gül, Hacı Ahmet Deveci, Ayla Deveci, Onur Akyıldırım, Mehmet Lütfi Yola

**Affiliations:** 1 Department of Nutrition and Dietetics, Faculty of Health Sciences, Mersin University, Mersin, 33343 Turkey; 2 Department of Nutrition and Dietetics, Faculty of Health Sciences, Gaziantep University, Gaziantep, 27000 Turkey; 3 Department of Property Protection and Security, Vocational School of Technical Sciences, Kilis7 Aralık University, Kilis, 79000 Turkey; 4 Department of Chemical Engineering, Faculty of Engineering and Architecture, Kafkas University, Kars, 36000 Turkey; 5 Department of Nutrition and Dietetics, Faculty of Health Sciences, Hasan Kalyoncu University, Gaziantep, 27000 Turkey

**Keywords:** Hydrazine, Square wave voltammetry, Molecularly imprinting, Nanocomposite, Tap water analysis

## Abstract

A hydrazine (HYD) detection method is presented based on molecularly imprinting polymers (MIPs) and cobalt-barium stannate nanoparticles incorporated-functionalized MWCNTs (CBSNPs/f-MWCNTs) nanocomposite. Firstly, co-precipitation and sonication techniques were applied to the preparations of CBSNPs and CBSNPs/f-MWCNTs nanocomposite, respectively. Since waste generation occurs at a minimum level in these techniques, an environmentally friendly nanocomposite was prepared. After the glassy carbon electrode modification with CBSNPs and CBSNPs/f-MWCNTs nanocomposite, HYD imprinted electrodes were fabricated using cyclic voltammetry (CV) with a dispersion containing 100.0 mM pyrrole (Py) monomer and 25.0 mM HYD molecule. The resulting electrochemical sensor demonstrated a detection span of 1.0 × 10^−9^ M to 1.0 × 10^−8^ M HYD and achieved a detection limit (LOD) of 3.0 × 10^−10^ M. Furthermore, the developed electrochemical sensor was used for actual tap water samples, and the obtained values close to 100.00% in recovery experiments showed the high accuracy of the developed sensor in real sample analysis. Finally, the selectivity, stability, and reproducibility of the created electrochemical sensor were investigated, and the developed sensor has been demonstrated to have high stability for at least 7 weeks, high reproducibility with a relative standard deviation (RSD) value of 0.14%, and high selectivity in real samples.

**Graphical Abstract:**

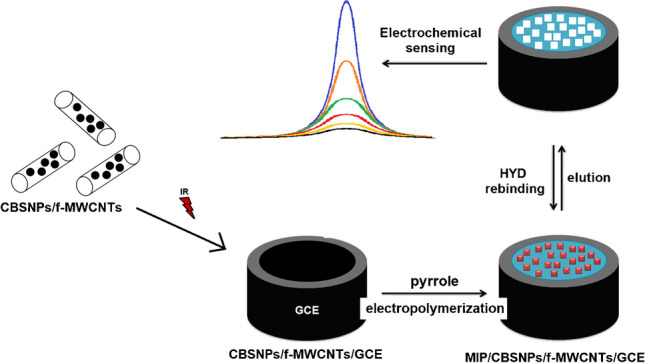

**Supplementary Information:**

The online version contains supplementary material available at 10.1007/s00604-025-06982-9.

## Introduction

HYD, a versatile and indispensable chemical, is a colorless, oily liquid with a sharp, ammonia-like odor, and an extremely reactive chemical compound with the formula N_2_H_4_. It is commonly utilized as a reducing agent, propellant fuel for rockets, and a precursor for numerous chemical reactions. Its ability to donate hydrogen atoms and act as a powerful reducing agent in chemical processes makes HYD valuable in many industries, from agriculture to pharmaceuticals [1, 2]. However, HYD’s extreme toxicity and instability present significant threats to both nature and human health. Therefore, accurately detecting and quantifying it in environmental samples, such as tap water, is crucial for public safety and regulatory compliance [3, 4]. Several analytical methods such as spectrophotometric method and gas chromatography have been published for HYD detection in air, water, and pharmaceutical samples [5–7]. However, when using these methods, special attention must be paid to equipment cleanliness to avoid cross-contamination. In addition, these approaches, often require complex sample preparation, are time-consuming, and require expensive equipment [8, 9]. These limitations prevent their widespread usage in routine monitoring and immediate on-site analysis. Hence, it is crucial to develop a sensor for HYD that is cost-effective, user-friendly, and robust, facilitating swift on-site analysis. In particular, sensors based on voltammetry and using a composite of nanomaterials are cheap, easy to manufacture, and highly sensitive; therefore, they have wide applications [10].

Recently, tin oxide nanostructures were investigated due to their thermal stability, and good catalytic activity. However, their some negative features including poor conductivity and valence band-edge can cause the low catalytic activity in electrochemical sensor applications [11, 12]. Particularly, perovskite stannate (ASnO_3_; A = Alkaline earth metals and transition metals) are significant nano-structured materials in terms of modern analytical applications due to a combination of qualities. In addition, these nano-structured materials exhibit strong redox behavior, good electronic conductivity, and fast electron transfer rate [13, 14]. Recently, trinary perovskite stannates containing three metal elements have gained importance as significant materials due to their enhanced performance resulting from the incorporation of metal elements into metal oxides. The trinary perovskite stannate has important applications such as photoreduction [15] and catalysts [16]. Additionally, CBSNPs have been widely employed in hydrogen evolution experiments, photocatalysis, and electrochemical analysis studies owing to their remarkable conductivity and chemical stability [17–19]. The existence of Co^2+^/Co^3+^ and Sn^2+^/Sn^4+^ contributes to the effective conductivity and high electrochemical catalytic properties [20]. Moreover, CBSNPs can provide the significant synergetic effect and available active sites resulting in the increase on the surface area for electrochemical sensor applications. In addition, the electrode surfaces can be modified with these nanoparticles to gain the impressive electrochemical results [21].

MWCNTs have been used extensively as sensor material in recent years owing to superior chemical properties, electrical conductivity, and large surface area [22, 23]. MWCNTs are utilized as sensor nanomaterial and can increase electron transfer in electrochemical processes. Nonetheless, insoluble pristine MWCNTs have negative situations such as self-aggregation between nano-structured tubes causing the low electrocatalytic performance. The MWCNTs functionalization with –COOH, and –OH groups in acidic medium can eliminate these limitations [24]. During MWCNTs functionalization, the oxygen-containing groups can incorporate into MWCNTs surfaces, providing better solubility, and the superior electrical conductivity causing the improved electrochemical catalytic activity [25, 26].

Compared with other identification systems, the molecular imprinting technology has many promising features such as selective molecular recognition, high affinity, and reusability. MIPs are used in various fields such as chromatography, purification, sensors, drug transport, and catalyst [27]. MIPs generally form a three-dimensional polymer network structure by copolymerization of appropriate functional monomers, cross-linker, and selected template molecule. In general, the MIP preparation mechanism occurs in three steps: (i) pre-complexation between appropriate functional monomers and the template molecule, (ii) three-dimensional polymer network formation in the presence of excess cross-linker, and (iii) After polymerization, the template molecule removal from the polymer to create specific cavities containing the size, shape, and functionality of the template molecule [28]. As a result, the synthesized imprinted polymer recognizes only the template molecule and binds selectively [29]. Metal oxide, MIPs, and carbon materials have been used to develop an electrochemical sensor including a double layer. Nonetheless, the usage of metal oxide materials can demonstrate a low conductivity and a low physical/chemical stability. Thus, the combination of metal oxide with MIPs can result in a remarkably electrochemical response. For example; a zinc oxide nanoparticles/MIP-based electrochemical sensor was presented for sodium dodecyl sulfate detection in laundry wastewater and shampoo samples. Though zinc oxide nanoparticles had good stability, the individual utilization of zinc oxide nanoparticles on electrode surface resulted in aggregation during the preparation step. Because of this, the combination of metal oxide with MIPs prevented the aggregation on electrode surface [30].

In this study, the high conductivity and stability properties of CBSNPs incorporated-functionalized MWCNTs nanocomposite and MIPs were combined at a high efficiency, and this type of electrochemical sensor was developed for the first time to be used in HYD analysis. Especially, the high selectivity, economic, and rapid preparation properties of MIPs provided high reproducibility and recovery in tap water samples. In addition, MIPs showed high catalytic activity with molecular recognition ability in comparison with other bio-catalytic systems [31, 32]. Notably, because direct modification of nanocomposite on the electrode surface caused high instability resulting from aggregation, the combination of metal oxide with MIPs provided stable electrochemical signals. Furthermore, the adherence to green chemistry principles during the sensor’s development including the nanocomposite synthesis and MIPs preparation suggested its environmentally friendly nature. In addition, because HYD analysis was performed with the square wave voltammetry (SWV) technique, which was a fast technique, HYD results could be monitored in real time. Ultimately, the functional utility of this sensor was demonstrated by detecting HYD in tap water samples, comparing its performance with standard analytical methods, and assessing its potential for broader environmental monitoring efforts.

## Experimental

### Chemicals and equipment

HYD, hydrogen peroxide (H_2_O_2_), uric acid (UA), ascorbic acid (AA), barium nitrate hexahydrate (Ba(NO_3_)_2_⋅6H_2_O, 99.90%), dopamine (DOP), cobalt nitrate hexahydride (Co(NO_3_)_2_⋅6H_2_O, ≥ 99.00%), sodium stannate (Na_2_[Sn(OH)_6_], 95.00%), multi-walled carbon nanotubes (MWCNTs, ≥ 99.00 wt%, 1.0–5.0 µm in length), Py monomer, phosphate buffer, and sodium chloride (NaCl) were procured by Sigma-Aldrich (USA).

Structural characterizations of CBSNPs, f-MWCNTs, and CBSNPs/f-MWCNTs nanocomposite were performed using the Bruker-Tensor Fourier transform infrared spectrometer (FTIR, Germany), scanning electron microscopy (SEM, ZEISS EVO 50 SEM, Tokyo, Japan), transmission electron microscopy (TEM, JEOL 2100 TEM, Tokyo, Japan), PHI 5000 Versa Probe type X-ray photoelectron spectroscopy (XPS, Japan/USA), and X-ray diffraction (XRD, Rikagu Miniflex X-ray diffractometer, Tokyo, Japan). The electrochemical measurements, including SWV, electrochemical impedance spectroscopy (EIS), and cyclic voltammetry (CV), were conducted through the Gamry Reference 600 workstation (USA).

### Preparation of f-MWCNTs and CBSNPs/f-MWCNTs nanocomposite

MWCNTs (0.40 g) were transferred into the mixture (10.0 mL) of HNO_3_-H_2_SO_4_ (3:1). After the dispersion was stirred at 100 °C for 20 h, the dispersion was dried at 40 °C for 15 h. Thus, MWCNTs were functionalized with –COOH groups (f-MWCNTs), providing the incorporation of CBSNPs.

A facile co-precipitation technique was utilized for the production of CBSNPs. The mixture of Ba(NO_3_)_2_⋅6H_2_O (2.0 mg), Co(NO_3_)_2_⋅6H_2_O (2.0 mg), and Na_2_[Sn(OH)_6_] (2.0 mg) was prepared in distilled water with stirring for 30 min. Following the addition of NaOH solution (1.0 mg mL^−1^) into the dispersion, the mixture was dried and subjected to heat treatment at 800 °C for 6 h, resulting in the formation of CBSNPs. A facile sonication method was used for the preparation of CBSNPs/f-MWCNT nanocomposite. For this aim, CBSNPs (3.0 mg) and f-MWCNTs (3.0 mg) were sonicated in dimethyl formamide for 30 min. Then, the dispersion was dried to produce CBSNPs/f-MWCNTs nanocomposite at 30 °C for 10 h [21].

### Development of CBSNPs/f-MWCNTs nanocomposite modified glassy carbon electrode (CBSNPs/f-MWCNTs/GCE)

The cleaning procedure for the glassy carbon electrode was carried out in accordance with the literature [33]. Then, the dropping treatment of the prepared CBSNPs/f-MWCNTs dispersion (20.00 µL, 1.00 mg mL^−1^) was performed on the GCE electrode (CBSNPs/f-MWCNTs/GCE). f-MWCNT-modified electrode (f-MWCNTs/GCE) was improved by using the same treatment including f-MWCNTs dispersion (20.00 µL, 1.00 mg mL^−1^).

### Preparation of HYD imprinted sensor and HYD removal

A high CV potential scan (from + 0.0 V to + 1.00 V) was conducted to an electrochemical cell including 100.0 mM Py monomer and 25.0 mM HYD molecule for the development of HYD imprinted CBSNPs/f-MWCNTs/GCE with cycle number of 20. Py was selected as monomer because it is a monomer with high conductivity, electropolymerization efficiency, and high physical inertness properties [34]. The number of scans reduced the signal levels of the detected polymerization peaks, which peaked at about + 0.70 V on the first scan. The observed peaks at the minimum peak current level indicated that the polymerization process was completed on the electrode surface (MIP/CBSNPs/f-MWCNTs/GCE).NIP/CBSNPs/f-MWCNTs/GCE was developed without the HYD molecule by repeating the above same procedure. NIP/CBSNPs/f-MWCNTs/GCE represented the prepared NIP electrode on the CBSNPs/f-MWCNTs/GCE electrode surface as a result of the above imprinting process without the target HYD molecule. MIP/bare GCE and MIP/f-MWCNTs/GCE were prepared using bare GCE in the presence of 100.0 mM Py monomer and 25.0 mM HYD molecule and f-MWCNTs/GCE in the presence of 100.0 mM Py monomer and 25.0 mM HYD molecule, respectively. The reference electrode and the counter electrode were a Ag/AgCl/KCl(sat) and a Pt wire, respectively.

For HYD removal, 1.0 M NaCl is commonly used to break hydrogen bonding/electrostatic interactions between monomer and analyte molecule in harmony with literature [35, 36]. For this aim, the MIP/CBSNPs/f-MWCNTs/GCE was put in a flask containing 1.0 M NaCl (10.0 mL) as desorption agent for the elimination of the hydrogen bonds (N–H⋯N) interactions between the Py monomer and HYD molecule at the desorption time of 20 min [37]. After that, the MIP electrode was dried under vacuum at 25 °C for subsequent analytical applications. In addition, the optimal analytical conditions for performing the analysis with the MIP were obtained as supporting electrolyte of pH 7.0 (1:4) of mole ratio between Py monomer and HYD molecule, the desorption time of 20 min and the CV scan cycle of 20. Finally, the frequency of 50 Hz, amplitude of 20 mV, and potential increment of 3 mV were applied to SWV measurements.

## Results and discussion

### Characterization of CBSNPs, f-MWCNTs, and CBSNPs/f-MWCNTs nanocomposite

**Fig. 1:**
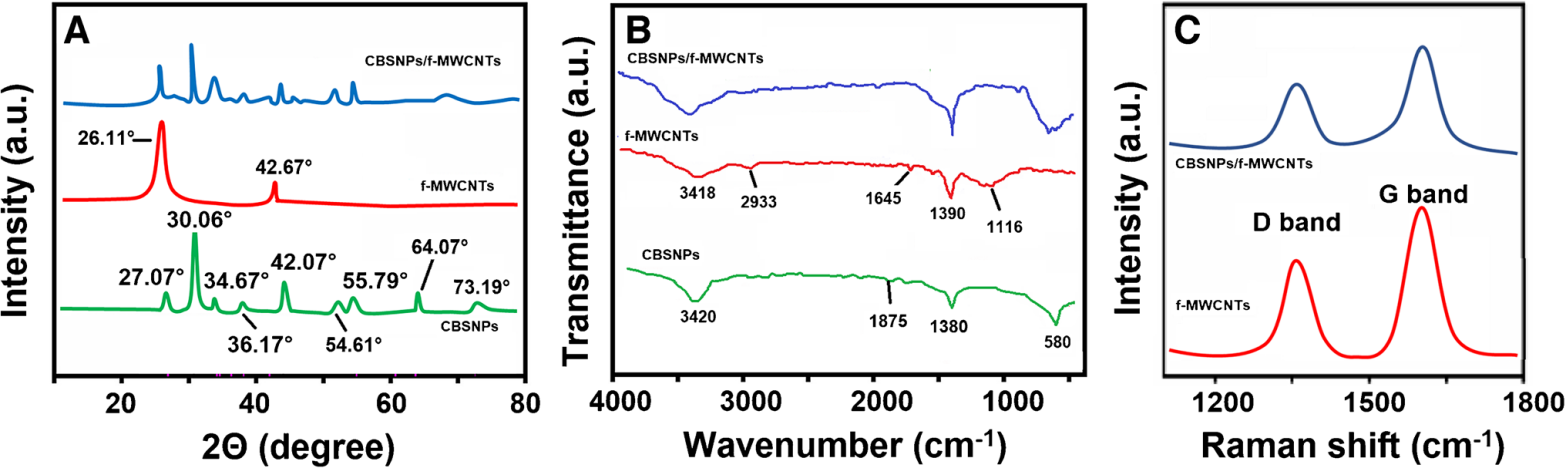
**A** XRD patterns, **B** FTIR spectra of CBSNPs, f-MWCNTs, and CBSNPs/f-MWCNTs nanocomposite, and **C** Raman spectra of f-MWCNTs and CBSNPs/f-MWCNTs nanocomposite

Figure 1A shows the crystal structure and physical phases of CBSNPs, f-MWCNTs, and the CBSNPs/f-MWCNTs nanocomposite as determined by XRD measurements. The XRD peaks belonging to CBSNPs at 27.07°(200), 30.06°(220), 34.67°(310), 36.17°(311), 42.07°(222), and 55.79°(420) were corresponded to CoSnO_3_ [38]. In addition, the XRD intensity peaks at 54.61°(211), 64.07°(220), and 73.19°(013) with major orientation along (110) plane were corresponded to cubic phase BaSnO_3_ [21]. Moreover, the XRD peaks at 26.11° and 42.67° were attributed to (002) and (100) planes of f-MWCNTs, respectively [39]. Subsequently, the XRD spectrum of CBSNPs/f-MWCNTs nanocomposite was in harmony with CBSNPs and f-MWCNTs, and the peaks belonging to CBSNPs/f-MWCNTs nanocomposite were slightly shifted, indicating the efficient interaction between CBSNPs and f-MWCNTs.

Figure 1B shows FTIR spectra of CBSNPs, f-MWCNTs, and CBSNPs/f-MWCNTs nanocomposite. For the FTIR spectrum of CBSNPs, the absorption band at 580 cm^−1^ was attributed to the metal–oxygen bonds’ vibrations [40]. Moreover, the –C–O and –C = O stretching modes were associated with the absorption bands at 1380 and 1875 cm^−1^ [41]. Furthermore, H–O–H and O–H stretching modes of absorbed H_2_O molecules were linked to the absorption band at 3420 cm^−1^ [42]. The FTIR peaks at 3418 and 2933 cm^−1^ were corresponded to –OH and –CH_2_– groups’ stretching vibrations belonging to f-MWCNTs [43]. Furthermore, the absorption bands at 1645, 1390, and 1116 cm^−1^ were attributed to C–C stretching and C–O bonds owing to –COOH group existence [44]. Thus, –COOH group attachment to MWCNTs surface was confirmed, and the specific absorption bands of CBSNPs/f-MWCNTs nanocomposite verified the successful interaction between CBSNPs and f-MWCNTs.

Figure 1C demonstrates Raman spectra of f-MWCNTs and CBSNPs/f-MWCNTs nanocomposite. The specific peaks of f-MWCNTs at 1360 and 1583 cm^−1^ were found to match with the graphitic carbon (represented by the D band) and the disordered carbon (represented by the G band), respectively. In the Raman spectrum of CBSNPs/f-MWCNTs nanocomposite, small shifts (1372 cm^−1^ at D band and 1586 cm^−1^ at G band) and the decreases in Raman peak intensities including G and D bands were observed, providing the interaction between CBSNPs and f-MWCNTs [21].

XPS investigations were also performed to determine the oxidation situation of the chemical elements (Fig. S1). According to the survey spectrum (Fig. S1A), the presence of Co2p, Ba3d, O1s, and C1s verified the successful preparation of CBSNPs/f-MWCNTs nanocomposite. Figure S1B exhibits the overlapping peaks belonging to Co2p and Ba3d and XPS peaks at 781.17 and 797.27 eV were attributed to Co2p_3/2_ and Co2p_1/2_ [45]. XPS peaks at 780.14 and 795.09 eV corresponding to Ba3d_5/2_ and Ba3d_3/2_ confirmed the presence of Ba(II) state [46]. Sn3d XPS spectrum exhibited four peaks (Fig. S1C). The two XPS peaks at 485.19 and 492.83 eV were assigned to Sn^2+^ of Sn3d_3/2_, and the two XPS peaks at 487.19 and 494.27 eV were corresponded to Sn^4+^ of Sn3d_5/2_ [47]. O1s peak consisted of three components and three XPS peaks at 530.09, 531.97, and 533.29 eV were attributed to the metal–oxygen bond (O1), the vacancy of oxygen (O2), and H_2_O molecule (O3) in the nanocomposite (Fig. S1D) for O1s spectra [48]. For C1s spectra, XPS peaks at 284.19 eV relating to –C–C/C = C–, 286.07 eV corresponding to –C–O and 287.49 eV attributing to –COOH, respectively, were observed (Fig. S1E) [49]. We can say that XPS results verified the successful synthesis of CBSNPs/f-MWCNTs nanocomposite.

**Fig. 2:**
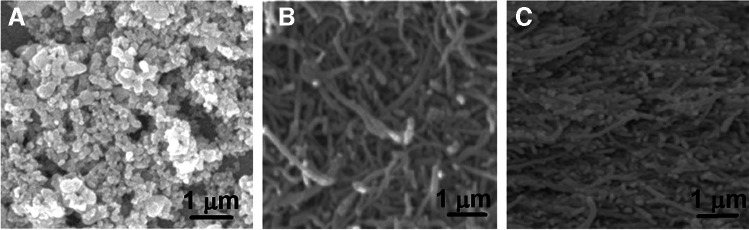
SEM images **A** CBSNPs, **B** f-MWCNTs, and **C** CBSNPs/f-MWCNTs nanocomposite

The surface morphology characteristics of CBSNPs, f-MWCNTs, and the CBSNPs/f-MWCNTs nanocomposite were examined through SEM images. According to Fig. 2A and 2, the nanostructured regular morphological structure of CBSNPs and the irregular morphological structure of MWCNTs were observed. In addition, Fig. 2C confirms the uniform incorporation of CBSNPs into f-MWCNTs and the synergistic interaction between CBSNPs and f-MWCNTs. In addition, according to EDS analysis (Fig. S2) of CBSNPs and CBSNPs/f-MWCNTs nanocomposite, a well-defined distribution of cobalt, barium, tin, and oxygen elements was observed on CBSNPs and CBSNPs/f-MWCNTs nanocomposite. Finally, TEM image (Fig. S3) of CBSNPs/f-MWCNTs nanocomposite was obtained in harmony with SEM image, indicating the CBSNPs embedded on f-MWCNTs [21]. We combined the usage of the software, ImageJ, with Origin to analyze TEM image. The area option was selected to measure the square of particles. Then, the diameter of the nanoparticle was synchronously mapped into the parameter of ImageJ, and the photo was magnified in the scale bar section. After that, a straight line was drawn with the same length as the scale bar (100 nm) [50]. Finally, the mean particle diameters of CBSNPs and f-MWCNTs were obtained as 12–15 nm and 40–45 nm, respectively.

### Electrochemical characterizations of f-MWCNTs and CBSNPs/f-MWCNTs nanocomposite–modified electrodes

**Fig. 3:**
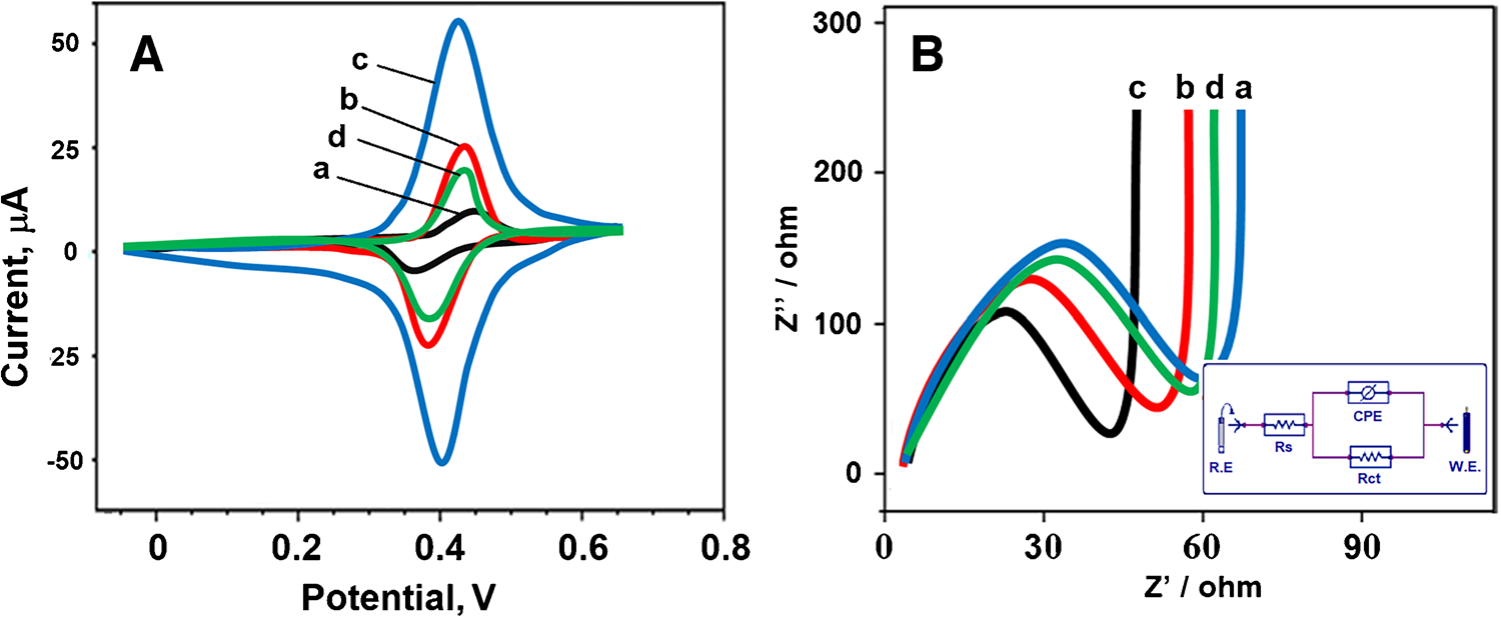
**A** CV curves and EIS responses at (a) bare GCE, (b) f-MWCNTs/GCE, (c) CBSNPs/f-MWCNTs/GCE, (d) MIP/CBSNPs/f-MWCNTs/GCE after the desorption of HYD molecules (Redox probe. 5.0 mM [Fe(CN)_6_]^3−/4−^ containing 0.1 M KCl; potential scan rate, 100 mV s.^−1^; inset was the Randles equivalent circuit for MIP/CBSNPs/f-MWCNTs/GCE, frequency range is 100,000–0.1 Hz with 10 mV wave amplitude at a formal potential of 0.150 V for EIS measurements, R.E stands for reference electrode and W.E for working electrode)

The electrochemical results of f-MWCNTs and CBSNPs/f-MWCNTs nanocomposite–modified electrodes and MIP/CBSNPs/f-MWCNTs/GCE after the desorption of HYD molecules were investigated by using CV (Fig. 3A) and EIS measurements (Fig. 3B). The anodic and cathodic peaks observed with the bare GCE electrode (curve a) became more prominent and catalyzed when f-MWCNTs/GCE (curve b) was used, due to carbon nanotubes’ superior structural, electrical, and electrochemical properties [51, 52]. The more pronounced electrochemical peaks were observed by using CBSNPs/f-MWCNTs/GCE (curve c) owing to perovskite stannate’s excellent properties such as electronic conducting properties and electron-transfer rate [53] and trinary perovskite stannate’s active sites and the large surface area [13]. Lastly, the fact that MIP/CBSNPs/f-MWCNTs/GCE after the desorption of HYD molecules (curve d) showed obvious anodic and cathodic peak currents towards 5.0 mM [Fe(CN)_6_]^3−/4−^ redox probe. The appearance of anodic and cathodic peaks with lower intensity on MIP/CBSNPs/f-MWCNTs/GCE in comparison with other electrodes such as f-MWCNTs/GCE and CBSNPs/f-MWCNTs/GCE proved the formation of the selective polymeric film. Notably, the development of a highly reproducible MIP-based electrochemical sensor is achieved by the regular binding capacities and immobilization on the electrode’s surface. These regular binding capacities and immobilization are sometimes negatively affected by non-specific interactions between the multifunctional nanocomposite material and the monomer on the electrode surface and the formed thick polymeric layer. It is possible to say that the observed lower electrochemical signals on MIP electrode occur due to the reasons explained above. However, because the obtained electrochemical signals had high selectivity and stability in this study, MIP/CBSNPs/f-MWCNTs/GCE electrode was used in real tap water samples [54].

Additionally, EIS measurements were performed to validate the CV results. Figure 3B shows the impedance plot (Nyquist diagram) of bare GCE, f-MWCNTs/GCE, CBSNPs/f-MWCNTs/GCE, and MIP/CBSNPs/f-MWCNTs/GCE after the desorption of HYD molecules. In addition, the inset of Fig. 3B demonstrated the experimental data fitting to the standard Randles equivalent circuit, which explained the solution resistance (R_s_), the charge transfer resistance (R_ct_), and the constant phase element (CPE). The experimental impedance values were matched with Randles equivalent circuit simulation using Gamry EIS 600 software. As shown in Fig. 3B, R_ct_ values were calculated as 60 Ω for bare GCE, 50 Ω for MIP/CBSNPs/f-MWCNTs/GCE after the desorption of HYD, 45 Ω for f-MWCNTs/GCE, and 40 Ω for CBSNPs/f-MWCNTs/GCE. Thus, these EIS results suggested the high availability of CBSNPs/f-MWCNTs nanocomposite in electrochemical sensor utility.

**Fig. 4:**
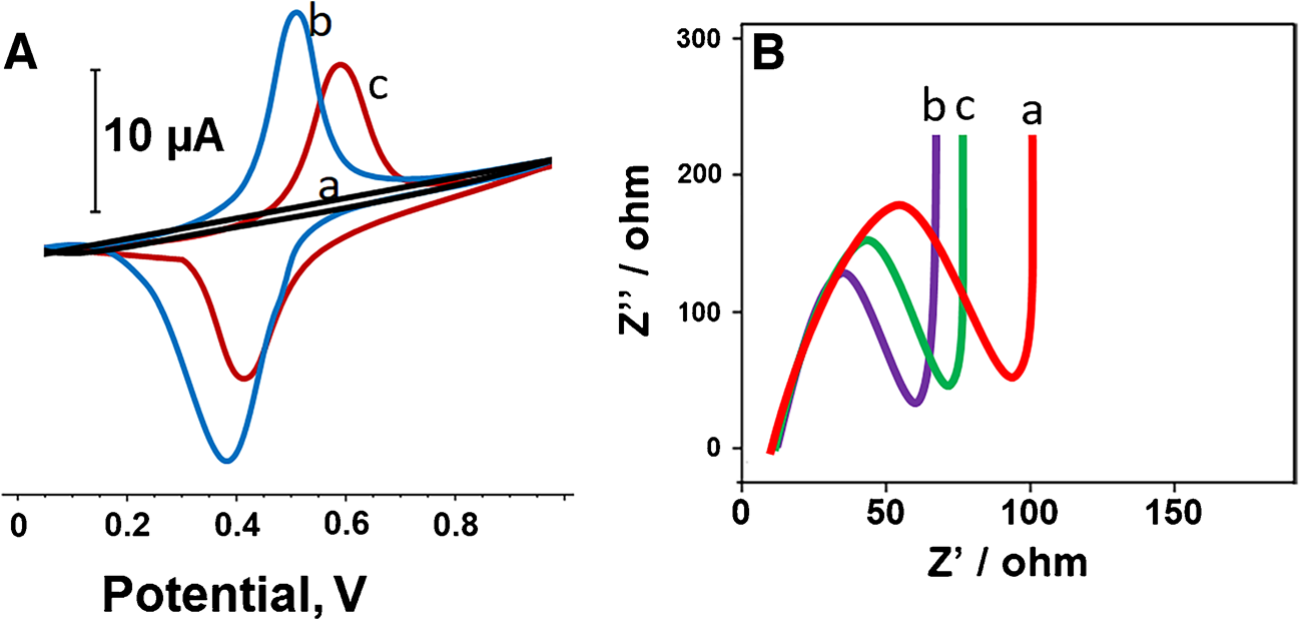
**A** CV curves and **B** EIS responses at (a) MIP/CBSNPs/f-MWCNTs/GCE without HYD removal, (b) MIP/CBSNPs/f-MWCNTs/GCE after the desorption of HYD molecules, and (c) NIP/CBSNPs/f-MWCNTs/GCE after the rebinding of HYD molecules (Redox probe, 5.0 mM [Fe(CN)_6_]^3−/4−^ containing 0.1 M KCl; potential scan rate, 100 mV s.^−1^, frequency range is 100,000–0.1 Hz with 10 mV wave amplitude at a formal potential of 0.150 V for EIS measurements)

Lastly, the electrochemical activities of MIP and NIP electrodes were investigated by using CV and EIS methods. According to Fig. 4A, the blockage of oxidation and reduction peaks belonging to redox probe was owing to Py formation (curve a) [55]. After the desorption of HYD molecules, a distinct peak current was observed because the electrochemically active regions emerged (curve b). In addition, when NIP/CBSNPs/f-MWCNTs/GCE was used with the rebinding of HYD molecules (curve c), as expected, a decrease in peak currents and distortions in peak shapes occurred. According to EIS measurements belonging to MIP and NIP electrodes (Fig. 4B), the lowest R_ct_ value was obtained by using MIP/CBSNPs/f-MWCNTs/GCE after the desorption of HYD molecules in harmony with Fig. 4A results, and the highest R_ct_ value was observed by using MIP/CBSNPs/f-MWCNTs/GCE without HYD removal in harmony with Fig. 4A. It has been proven that electron transfer was accelerated on the electrode surface by the emergence of electroactive sites by removing the target molecule HYD from the electrode surface.

### Development of HYD imprinted polymer on CBSNPs/f-MWCNTs/GCE and the mechanism for HYD imprinted polymer formation

**Fig. 5:**
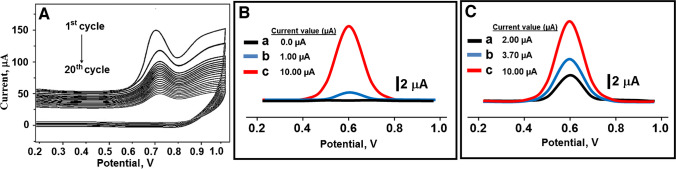
**A** CV polymerization on CBSNPs/f-MWCNTs/GCE (potential scan rate, 100 mV s^−1^). **B** The square wave voltammograms of (a) MIP/CBSNPs/f-MWCNTs/GCE in phosphate buffer (pH 7.0), (b) NIP/CBSNPs/f-MWCNTs/GCE in the presence of 10.0 nM HYD in 0.1 M phosphate buffer (pH 7.0), and (c) MIP/CBSNPs/f-MWCNTs/GCE in presence of 10.0 nM HYD in 0.1 M phosphate buffer (pH 7.0). **C** The square wave voltammograms in presence of 10.0 nM HYD in 0.1 M phosphate buffer (pH 7.0) of (a) MIP/bare GCE, (b) MIP/f-MWCNTs/GCE, and (c) MIP/CBSNPs/f-MWCNTs/GCE

Using CBSNPs/f-MWCNTs/GCE as the working electrode, a potential of up to + 1.00 V was imposed on the mixture containing 100.0 mM Py monomer and 25.0 mM analyte molecules in an electrochemical cell. The electrochemical signal values (µA) of the polymerization peaks observed around + 0.70 V in the first scan decreased as the number of scans increased and reached their minimum value at the 20th scan number (Fig. 5A). This proved that the polymerization process was completed and the MIP-based electrode was prepared.

To show that the molecular imprinting technique provided a high imprinting selectivity in MIP-based sensors, MIP and NIP-based electrodes were prepared and tested in the presence of 10.0 nM HYD. Any electrochemical signals not observed without the analyte solution (curve a of Fig. 5B) were observed at 1.0 µA using the NIP electrode (curve b of Fig. 5B) and at 10.0 µA using the MIP-based electrode (curve c of Fig. 5B). This tenfold signal enhancement showed that the MIP-based electrode provided high selectivity for HYD detection. Finally, as expected, the electro-catalysis properties of the prepared MIP electrodes step by step were examined (Fig. 5C), and it was found that the MIP/CBSNPs/f-MWCNTs/GCE electrode showed the highest electrochemical signal against HYD. Furthermore, SEM images of MIP and NIP electrodes were obtained to confirm the formation of molecularly imprinting polymers. According to Fig. S4, it was observed that porous structures were formed in the MIP electrode; however, the NIP electrode was more non-porous. NIP electrode was uniform in size, and no cavities were generated. However, MIP electrode exhibited better site accessibility for special recognition toward the target analyte.

The mechanism including the targeted adsorption of HYD in MIP/CBSNPs/f-MWCNTs system was presented. Because there were no functional groups in HYD analyte providing strong intermolecular interactions with MIP/CBSNPs/f-MWCNTs, the interaction between HYD analyte and MIP/CBSNPs/f-MWCNTs during MIP formation was mainly noncovalent interactions, such as hydrogen bonding.

### Optimization

#### pH effect

The pH values of the support electrolyte employed in electrochemical sensor applications are regarded as the most critical element influencing the stability of the electrochemical sensor. In this study, the current signals (µA) against MIP/CBSNPs/f-MWCNTs/GCE electrode were measured by preparing a supporting electrolyte between pH 3.0 and 9.0 (Fig. S5A). If the pH value was lower than pKa (HYD) = 8.1, HYD was positively charged and the repulsion of HYD molecules from the electrode surface occurred. If the pH value approached the pKa value, the uncharged form of HYD molecules formed, providing an increase in the peak currents. However, the much increase on pH values caused a decrease in the apparent surface coverage, suggesting the reduction of electrochemical performance [56]. Thus, the stability of the formed interaction between monomer and analyte was at the maximum level in a neutral medium, and the highest peak current values were obtained using pH 7.0, 0.1 M phosphate buffer. In addition, the electrochemical detection mechanism relating to the oxidation in 0.1 M phosphate buffer (pH 7.0) was explained below in Eqs. (1)–(3) [57].

1 $$\begin{array}{ccc}N_2H_4+H_2O&\rightarrow&N_2H_3+H_3O^++e^-\;\left(slow-rate\right)\end{array}$$

2 $$\begin{array}{ccc}N_2H_3+3H_2O&\rightarrow&N_2+3H_3O^++3e^-\;\left(fast\;rate\right)\end{array}$$

3 $$\begin{array}{ccc}N_2H_4+4H_2O&\rightarrow&N_2+4H_3O^++4e^-\left(all\;reaction\right)\end{array}$$

#### Mole ratio effect

The primary element influencing sensor sensitivity in MIP-based electrochemical applications is the development of a thick or thin polymeric layer on the electrode surface. On the electrode surface, non-specific interactions happen, especially when the monomer ratio is maintained high. Conversely, unique interactions between the monomer and analyte molecules are infrequent when this ratio is low. The maximum sensitivity was observed in the prepared MIP/CBSNPs/f-MWCNTs/GCE electrode when the amount of monomer was 100.0 mM and the amount of analyte molecules was 25.0 mM (Fig. S5B).

#### Scan cycle effect

During this study, the scan numbers between 10 and 50 were tried to obtain the optimum CV scan number, and maximum electrochemical sensor signals were tried to be obtained. After 20 scan numbers, the formation of a thick polymeric film on the electrode surface decreased the sensor sensitivity. According to Fig. S5C, the scan cycle of 20 was selected during the preparation of the MIP-based electrode.

#### Desorption time effect

Another important factor affecting sensor sensitivity in MIP-based electrochemical sensor applications is desorption time. Specifically, the highest level of sensor sensitivity is achieved during the desorption stage when all analyte molecules are removed from the electrode surface. According to Fig. S5D, the desorption time of 20 min was selected during template molecule removal studies.

### Sensitivity of MIP/CBSNPs/f-MWCNTs/GCE sensor

**Fig. 6:**
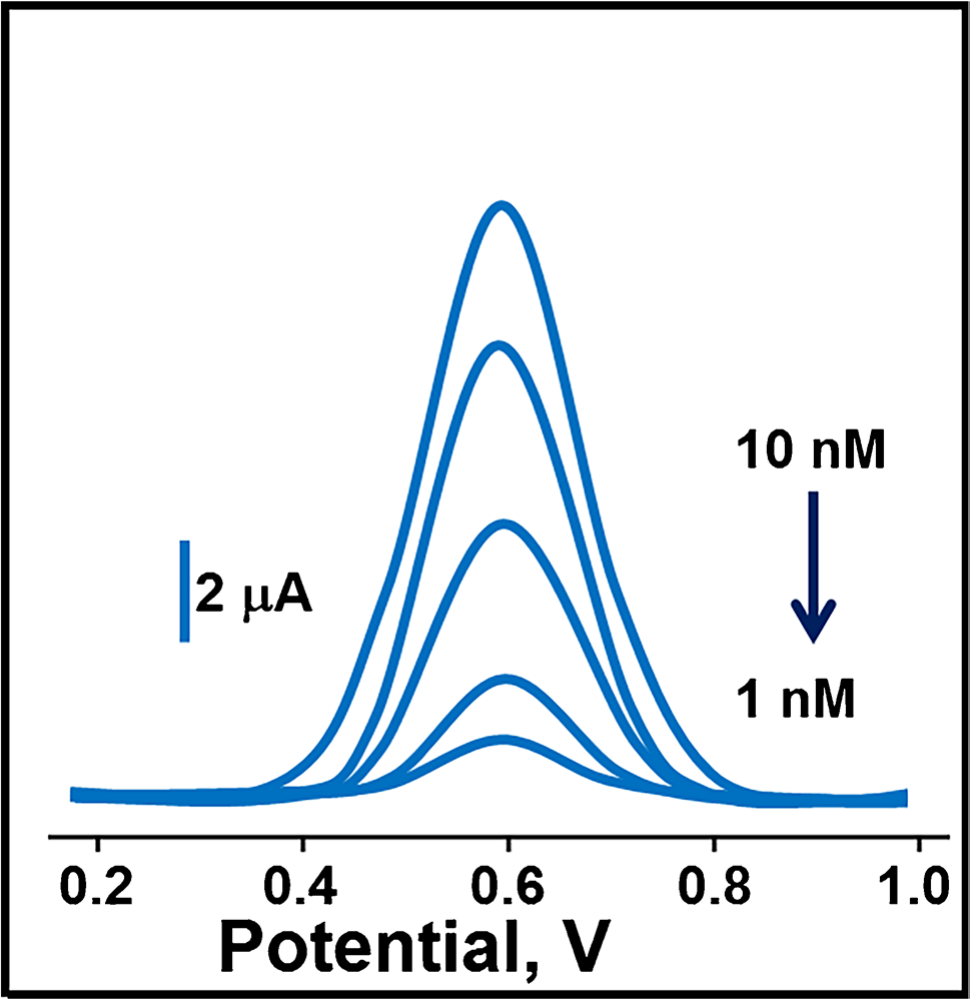
Square wave voltammograms at increasing HYD concentrations (from 1.0 to 10.0 nM) by using MIP/CBSNPs/f-MWCNTs/GCE

In the proposed study, the SWV method, one of the efficient electroanalytical techniques, was used for HYD analysis. In the SWV technique, the current occurring in an electrochemical cell under full-concentration polarization conditions is measured. Since this technique is an electroanalytical method performed under polarization conditions, micro electrodes are used as working electrodes. In recent years, conductive polymers and amalgam electrodes, pencil graphite electrodes, carbon paste electrodes, and modified electrodes obtained by modifying them with nanoparticles or some chemicals have been used in sensor applications [58–62]. In addition, it is a fast and sensitive electrochemical technique, and the entire voltammogram can be observed in 10 ms. In this study, the linearities between the current signals and HYD concentration were obtained as y (µA) = 1.1737*x* (C_HYD_, nM) − 0.2057 at MIP/CBSNPs/f-MWCNTs/GCE (Fig. 6) and *y* (µA) = 0.1174*x* (C_HYD_, nM) − 0.0206 at NIP/CBSNPs/f-MWCNTs/GCE (Fig. S6) in range from 1.0 × 10^−9^ M to 1.0 × 10^−8^ M HYD. According to Fig. S6, the current values obtained using the NIP electrode were observed to be 10 times less than the current values obtained using the MIP electrode in all HYD concentrations within the calibration range. The biggest reason for this difference can be explained as follows: When a molecular imprinting process was carried out without the target molecule HYD during the preparation of the NIP electrode, HYD-specific polymeric surfaces were not formed on the electrode surface. This situation affected the sensor sensitivity and caused the low HYD-specific sensor signals, confirming the high important application of molecularly imprinting technique. In addition, the reason for the low sensor signals on the NIP electrode surface was due to non-specific monomer-electrode interactions occurring on the electrode surface during the NIP electrode preparation. The limit of quantification (LOQ) and LOD values were 1.0 × 10^−9^ M and 3.0 × 10^−10^ M, respectively (see Supplementary Data for the equations)*.* Data of the calibration curves for the developed sensor were given in Table S1. Based on Table 1, the sensor developed in this study demonstrated a higher sensitivity in HYD analysis compared to the analytical methods proposed in recent literature. Thanks to the SWV method, which was a particularly fast technique; the current signals were recorded within 10 ms. Furthermore, during the synthesis of CBSNP incorporated-functionalized MWCNTs nanomaterial, a production technique with minimum waste generation and high efficiency was used in accordance with green chemistry, providing an environmentally friendly analysis technique.

**Table 1:** The comparison of MIP/CBSNPs/f-MWCNTs/GCE’s performance with the reported methods

Material/method	Linear range (M)	LOD (M)	Ref.
ZnO@TiO_2_NPs	1.0 × 10^−8^–5.85 × 10^−4^	5.0 × 10^−9^	[63]
C–CeO2 nanofibers	1.5 × 10^−8^–1.42 × 10^−3^	8.0 × 10^−9^	[64]
Cit-AuNPs	3.0 × 10^−6^–1.0 × 10^−3^	1.1 × 10^−8^	[65]
MoS_2_-QDs@Fe_3_O_4_/GCE	8.0 × 10^−7^–2.20 × 10^−3^	1.2 × 10^−7^	[66]
Nitrogen-doped hollow carbon spheres	2.0 × 10^−8^–3.80 × 10^−4^	7.0 × 10^−9^	[67]
Au nanoparticles decorated on the poly(Nile Blue)	1.0 × 10^−8^–1.90 × 10^−4^	3.3 × 10^−7^	[68]
MIP/CBSNPs/f-MWCNTs/GCE	1.0 × 10^−9^–1.0 × 10^−8^	3.0 × 10^−10^	This study

### Recovery

Recovery experiments were performed to demonstrate the applicability of the highly selective HYD sensor in real sample conditions. For this purpose, after the tap water samples (1.0 mL) were firstly filtered with a 1.0-µm filter, the tap water samples were diluted with 0.1 M, pH 7.0 phosphate buffer to fall within the linearity range. Then, HYD standard solutions with increasing concentrations (2.00, 4.00, and 6.00 nM HYD) were added into the divided tap water samples into equal volumes, except for the first tap water sample, and then all tap water samples were diluted with 0.1 M phosphate buffer. According to the close values to 100.00%, the developed sensor within the scope of this study has been proven to be used with a high level of verification (Table 2).

**Table 2:** Recovery results of HYD (*n* = 6)

Sample	Added HYD (nM)	Found HYD (nM)	RSD (%)	*Recovery (%)
Tap water	-	0.390 ± 0.001	0.63	-
	2.000	2.400 ± 0.006	0.10	100.42 ± 0.04
	4.000	4.380 ± 0.003	0.17	99.77 ± 0.06
	6.000	6.380 ± 0.007	0.27	99.84 ± 0.03

*Recovery = Found HYD, nM / Real HYD, nM; *RSD* % relative standard deviation

LC–MS/MS was also utilized for the evaluation of the validity of the developed sensor in this study [69]. According to the obtained analysis results from both techniques (Table 3), there was no important difference between the developed sensor in this study and LC–MS/MS (*T*_calculated_ > *T*_tabulated_, *p* > 0.05).

**Table 3:** Comparison of the results by the developed sensor in this study and LC–MS/MS for HYD detection (n = 6) (Added HYD = 2.000 nM)

	Found HYD	
Sample	MIP/CBSNPs/f-MWCNTs/GCE	LC–MS/MS
Tap water (nM)	2.400 ± 0.006	2.401 ± 0.007
RSD	0.61	0.71

$\overline X$: Mean ± Standard Error; RSD: % Relative Standard Deviation

### Selectivity, stability, and reproducibility of MIP/CBSNPs/f-MWCNTs/GCE sensor

**Fig. 7:**
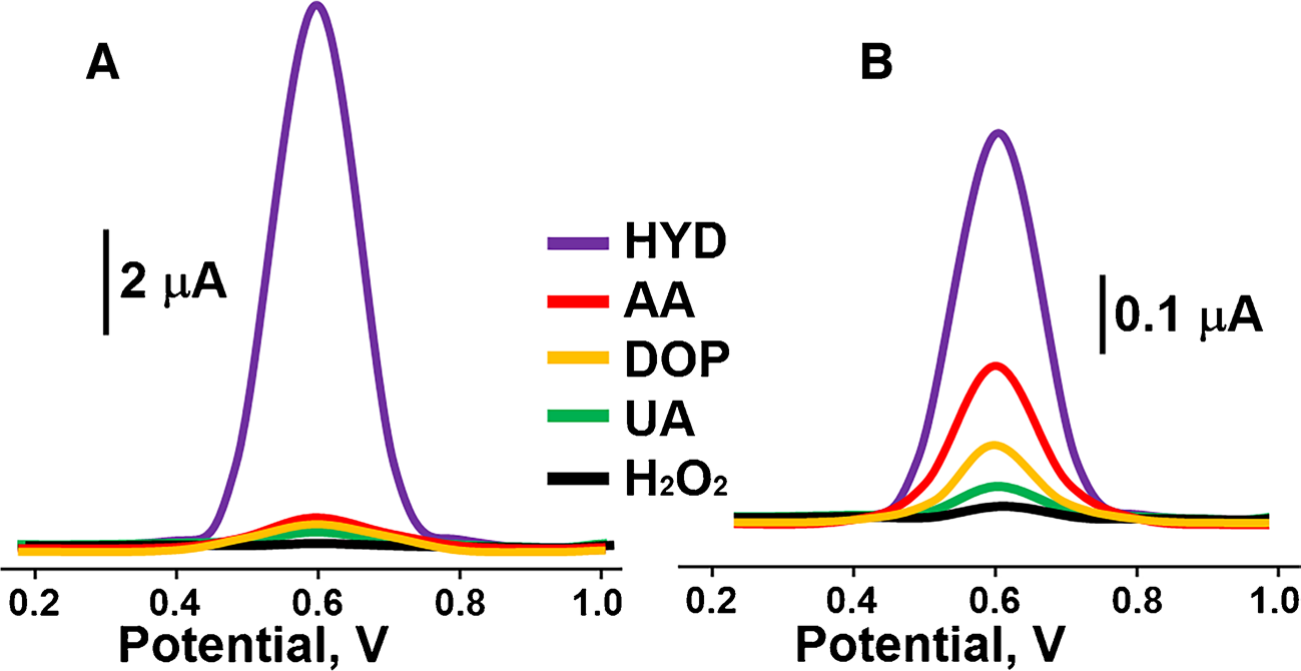
Selectivity tests: Square wave voltammograms of **A** MIP/CBSNPs/f-MWCNTs/GCE and **B** NIP/CBSNPs/f-MWCNTs/GCE in 10.0 nM HYD, 1000.0 nM AA, 1000.0 nM DOP, 1000.0 nM UA, and 1000.0 nM H_2_O_2_

Figure 7A and 7 indicates the square wave voltammograms attributing to MIP/CBSNPs/f-MWCNTs/GCE and NIP/CBSNPs/f-MWCNTs/GCE in the presence of 10.0 nM HYD, 1000.0 nM AA, 1000.0 nM DOP, 1000.0 nM UA, and 1000.0 nM H_2_O_2_. Table 4 shows that MIP electrode was 21.00, 26.25, 35.00, and 105.00 times more selective for HYD than AA, DOP, UA, and H_2_O_2_, respectively, because of the prepared HYD selective nano-cavieties in electrode surfaces. Thus, it is possible to say that molecular imprinting technology provides an important advantage such as high selectivity in target molecule detections in real sample environments.

**Table 4:** k and k′ values of MIP/CBSNPs/f-MWCNTs/GCE and NIP/CBSNPs/f-MWCNTs/GCE (n=6)

	MIP		NIP		
	∆i	k	∆i	k	k′
HYD	10.50 ± 0.03	-	0.50 ± 0.02	-	-
AA	0.50 ± 0.04	21.00	0.20 ± 0.01	2.50	8.40
DOP	0.40 ± 0.02	26.25	0.10 ± 0.04	5.00	5.25
UA	0.30 ± 0.01	35.00	0.05 ± 0.07	10.00	3.50
H_2_O_2_	0.10 ± 0.04	105.00	0.01 ± 0.06	50.00	2.10

Analyte concentrations: 10.0 nM HYD, 1000.0 nM AA, 1000.0 nM DOP, 1000.0 nM UA, 1000.0 nM H_2_O_2_k (selectivity coefficient) = ∆i_HYD_/∆i_interfering chemical_ and k′ (relative selectivity coefficient) = k_MIP/_k_NIP_

The stability of the prepared MIP/CBSNPs/f-MWCNTs/GCE electrode was assessed by measuring the peak currents in the presence of 10.0 nM HYD over a period of 7 weeks. At the conclusion of the 7th week, the average peak current signal (μA) was approximately 99.02% of that recorded at the end of the first week, demonstrating significant stability (Fig. S7).

The reproducibility test for the prepared MIP/CBSNPs/f-MWCNTs/GCE electrode involved measuring the obtained peak currents (µA) from 40 HYD imprinted electrodes in the presence of 10.0 nM HYD. The obtained peak currents (µA) from 40 HYD imprinted electrodes exhibited a RSD value of 0.14%, indicating high reproducibility.

## Conclusions

To sum up, an innovative electrochemical sensor for HYD imprinting was developed by utilizing CBSNPs/f-MWCNT nanocomposite. After the preparation of the CBSNPs/f-MWCNT nanocomposite with high impurity by using an environmentally friendly technique, the developed sensor demonstrated a linearity of 1.0 × 10^−9^–1.0 × 10^−8^ M HYD with a sensitive LOD of 3.0 × 10^−10^ M. The sensor was applied to tap water samples with the recovery values close to 100.0%. LC–MS/MS was also used for the evaluation of the high validity of the developed sensor, and no significant difference was found between the results (*T*_calculated_ > *T*_tabulated_, *p* > 0.05). Finally, the developed sensor was evaluated in terms of selectivity, stability, and reproducibility.

## Supplementary information

Below is the link to the electronic supplementary material.

ESM 1 (DOC 1.30 MB)

## Data Availability

No data sets were generated or analyzed during the current study.
