# Supplementary material for: Hydrazine imprinted electrochemical sensor based on cobalt-barium stannate nanoparticles incorporated-functionalized MWCNTs nanocomposite for hydrazine determination in tap water samples
Source: Mikrochim Acta. 2025 Feb 1;192(2):124. doi: 10.1007/s00604-025-06982-9 (PMC11787222; doi:10.1007/s00604-025-06982-9)
Supplement: Supplementary file 1 — (DOC 1.30 MB) [file 604_2025_6982_MOESM1_ESM.doc]

**Supplementary Data For**

**Hydrazine imprinted electrochemical sensor based on cobalt-barium stannate nanoparticles incorporated-functionalized MWCNTs nanocomposite for hydrazine determination in tap water samples**

Fatma Hazan Gül^1^, Hacı Ahmet Deveci^2^, Ayla Deveci^3^, Onur Akyıldırım^4^, Mehmet Lütfi Yola^5^*

^1^Department of Nutrition and Dietetics, Faculty of Health Sciences, Mersin University, Mersin, 33343, Turkey; [fatmagul@mersin.edu.tr](mailto:fatmagul@mersin.edu.tr)

^2^Department of Nutrition and Dietetics, Faculty of Health Sciences, Gaziantep University, Gaziantep, 27000, Turkey; h_ahmet_deveci@gantep.edu.tr

^3^Department of Property Protection and Security, Vocational School of Technical Sciences, Kilis 7 Aralık University, Kilis, 79000, Turkey; ayladeveci@kilis.edu.tr

^4^Department of Chemical Engineering, Faculty of Engineering and Architecture, Kafkas University, Kars, 36000, Turkey; onurakyildirim@gmail.com

^5^Department of Nutrition and Dietetics, Faculty of Health Sciences, Hasan Kalyoncu University, Gaziantep, 27000, Turkey

*****Correspondence: mlutfi.yola@hku.edu.tr; Tel.: +90-3422118080; Fax: +90-3422118081

**Linearity**

*LOQ = 10.0 S / m*

*LOD = 3.3 S / m*

S: Standard deviation of the intercept and m*:* Slope of the regression line

**Table S1.** Data of the calibration curves for the proposed sensor (n = 6)

| Regression equation | y (µA) = 1.1737x (C_HYD_, nM) - 0.2057 |
| --- | --- |
| Standard error of slope | 0.0018 |
| Standard error of intercept | 0.0011 |
| Correlation coefficient (r) | 0.9996 |
| Linearity range (M) | 1.0×10^-9^ – 1.0×10^-8^ |
| Number of data points | 5 |
| LOD (M) | 3.0×10^-10^ |
| LOQ (M) | 1.0×10^-9^ |

**
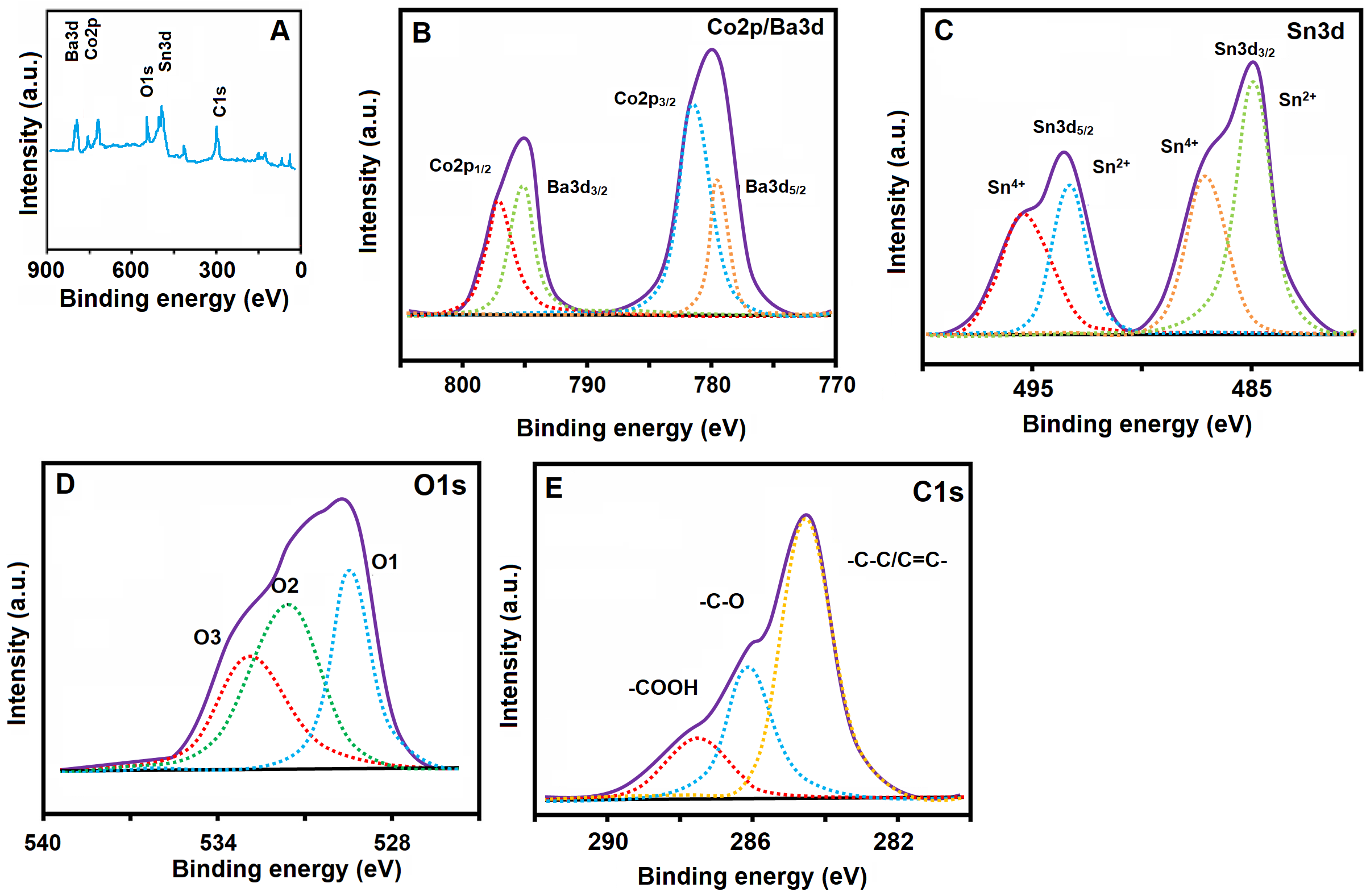
Fig. S1.** (A) XPS survey spectrum of CBSNPs/f-MWCNTs nanocomposite, the high-resolution XPS spectra of (B) Co2p/Ba3d, (C) Sn3d, (D) O1s, and (E) C1s

**
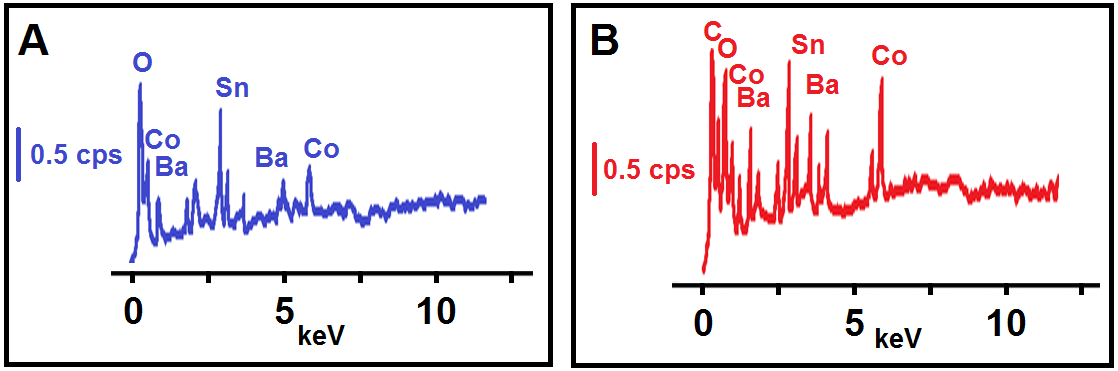
Fig. S2.** EDS analysis (A) CBSNPs and (B) CBSNPs/f-MWCNTs nanocomposite

**
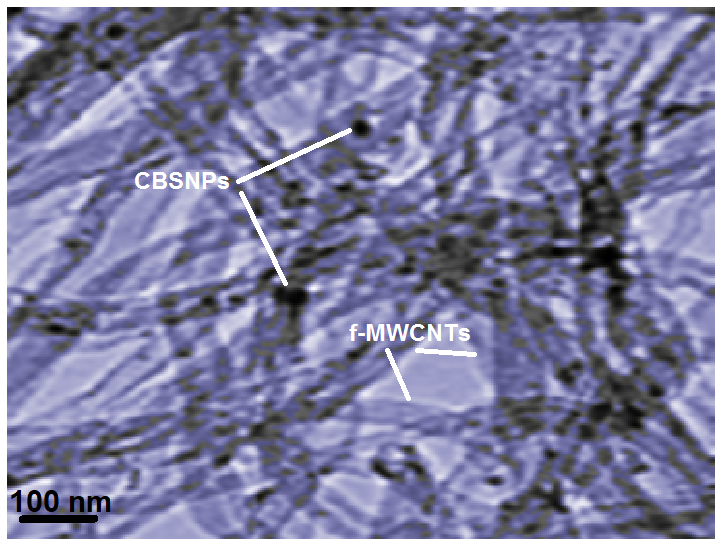
**

**Fig. S3.** TEM image of CBSNPs/f-MWCNTs nanocomposite


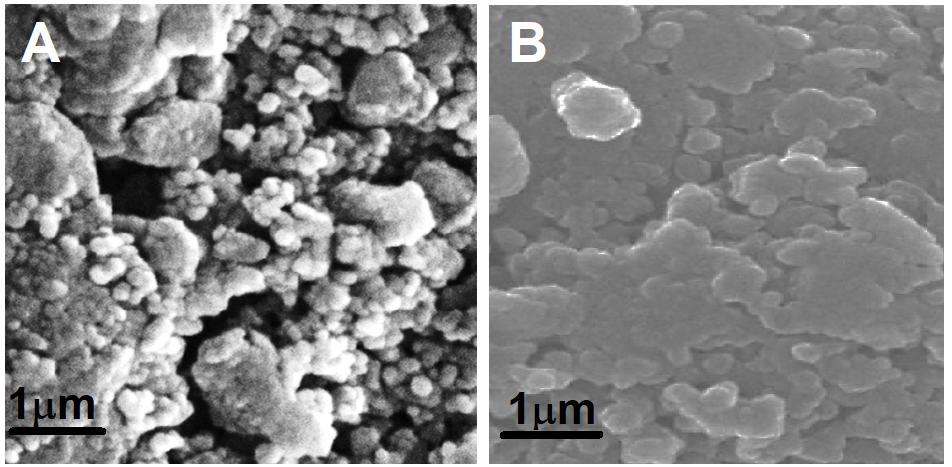


**Fig. S4**. SEM images (A) MIP/CBSNPs/f-MWCNTs/GCE and (B) NIP/CBSNPs/f-MWCNTs/GCE

**
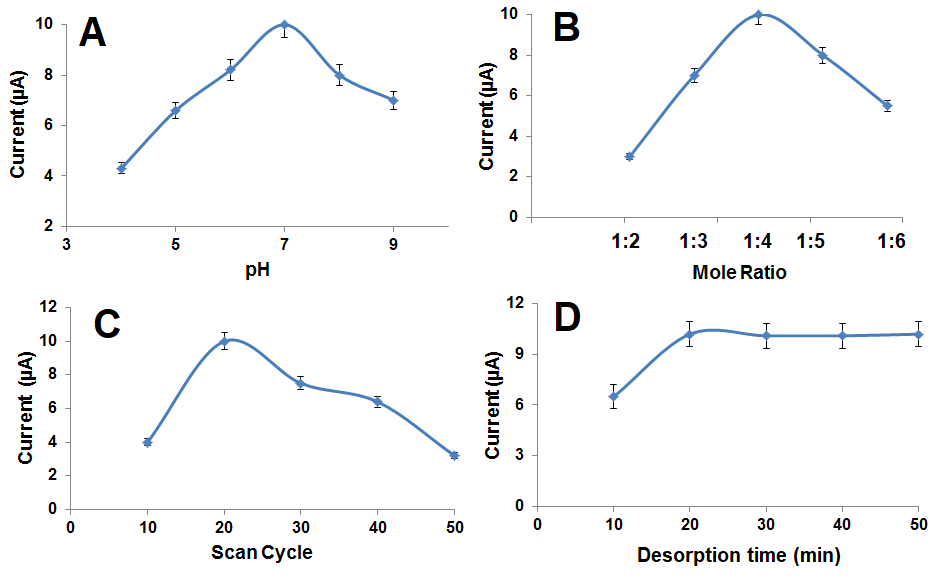
Fig. S5.** Effect of (A) pH, (B) mole ratio, (C) scan cycle, (D) desorption time on signals of SWVs in presence of 10.0 nM HYD in 0.1 M, pH 7.0 phosphate buffer (n=6)

**
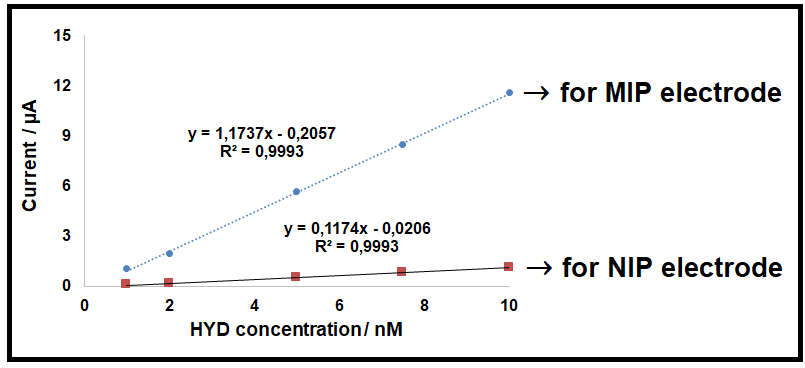
Fig. S6.** Calibration curves of HYD concentrations at MIP/CBSNPs/f-MWCNTs/GCE and NIP/CBSNPs/f-MWCNTs/GCE

**
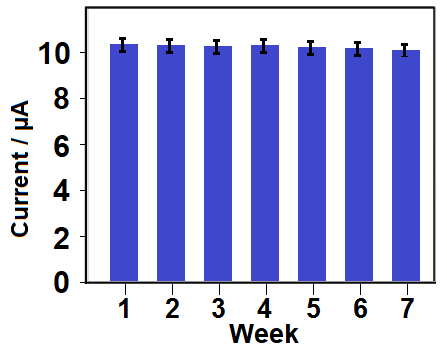
**

**Fig. S7.** Stability test of MIP/CBSNPs/f-MWCNTs/GCE in presence of 10.0 nM HYD in 0.1 M, pH 7.0 phosphate buffer (n = 6)
